# STELLAR: fast and exact local alignments

**DOI:** 10.1186/1471-2105-12-S9-S15

**Published:** 2011-10-05

**Authors:** Birte Kehr, David Weese, Knut Reinert

**Affiliations:** 1 Department of Computer Science, Free University Berlin, Takustr. 9, 14195 Berlin, Germany; 2 International Max Planck Research School for Computational Biology and Scientific Computing, Ihnestr. 63-73, 14195 Berlin, Germany

## Abstract

**Background:**

Large-scale comparison of genomic sequences requires reliable tools for the search of local alignments. Practical local aligners are in general fast, but heuristic, and hence sometimes miss significant matches.

**Results:**

We present here the local pairwise aligner STELLAR that has full sensitivity for *ε*-alignments, i.e. guarantees to report all local alignments of a given minimal length and maximal error rate. The aligner is composed of two steps, filtering and verification. We apply the SWIFT algorithm for lossless filtering, and have developed a new verification strategy that we prove to be exact. Our results on simulated and real genomic data confirm and quantify the conjecture that heuristic tools like BLAST or BLAT miss a large percentage of significant local alignments.

**Conclusions:**

STELLAR is very practical and fast on very long sequences which makes it a suitable new tool for finding local alignments between genomic sequences under the edit distance model. Binaries are freely available for Linux, Windows, and Mac OS X at http://www.seqan.de/projects/stellar. The source code is freely distributed with the SeqAn C++ library version 1.3 and later at http://www.seqan.de.

## Introduction

Computing good local alignments is a fundamental problem in bioinformatics. By looking for local alignments of biological sequences one aims for example at identifying homologous regions, i.e. regions that are assumed to originate from the same ancestral sequence, or at finding functionally similar sequences. The problem has been studied for more than 30 years [1,2], but still remains interesting. In the beginning, local alignments were used to look for homologous regions in relatively short protein or nucleic acid sequences. Also, for a long time, local alignments have been used to identify conserved, functionally related elements. More recently, local alignments were applied on a genomic scale as prerequisite to global genomic alignments. For several reasons genomic scale alignments are usually not collinear and hence one has to resort to computing local similarities. Now the aim is not anymore to identify *some* homologous regions but rather to display *all* similarities between two or more genomic sequences [3-7]. This requires not only efficiency in computation to process very long sequences, but also accuracy regarding sensitivity, i.e. exact tools that do not miss regions of significant local similarity.

For the computation of local alignments numerous tools have been developed: Early tools such as SSEARCH [2] and FASTA [16] are sensitive but too slow for large-scale sequence comparison. Then, there are efficient heuristics, with the BLAST family [17-19] being the most prominent example. Further developments for specific large-scale analyzes resulted in tools like BLAT [20] which was designed for high speed, and BLASTZ [21] which was designed for higher sensitivity. The more recently published tool BWT-SW [22] again focuses on sensitivity and is able to report all local alignments.

To assess homology of biological sequences by local alignment, generally some kind of similarity criterion is necessary. The most widely accepted criterion is the *E-value*[18,23], a probabilistic measure that assesses the significance of a local alignment. The E-value denotes the expected number of local alignments with a minimal score occurring by chance in the input sequences. The score underlying the E-value is a Smith-Waterman-like score, most commonly using affine gap costs. All popular alignment tools that report E-values, e.g. BLAST, compute such a score, and afterward apply an E-value threshold on their output. In this paper, we describe a method that follows an alternative approach to compute only significant local alignments of nucleic acid sequences: We compute only high-scoring alignments, which are guaranteed to have a good (low) E-value. We use a maximal error rate for local alignments (normalized edit distance) as a score threshold, and additionally require a minimal alignment length. Our method is specialized on relatively low error rates, which in turn justifies the use of edit distance instead of affine gap costs. For a given error rate and a minimal alignment length our method is exact, i.e. it identifies all local alignments without loss of sensitivity. We compute an E-value for all generated local alignments and a minimal E-value for the input parameters. The method is implemented in the program STELLAR (SwifT Exact LocaL AligneR) using the SeqAn C++ library [24,25]. The program depends only on very few and clearly understandable parameters. We prove that our algorithm is exact for all reasonable parameter settings and confirm this experimentally. We compare STELLAR against popular local alignment programs, namely BLAST, LASTZ, BLAT, and BWT-SW in terms of speed and sensitivity and show that some of the tools miss many significant local alignments that can be detected with STELLAR.

## Methods

### Definitions and overview of algorithm

**Figure 1:**
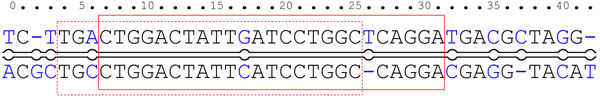
**Overlapping *ε*-matches.** An alignment of two strings containing two overlapping *ε*-matches for *ε* = 0.1 and *n*_0_ = 20. The *ε*-match indicated by the dashed box has an error rate of 2/22, the *ε*-match indicated by the box with a continuous line one of 2/25. The union of these two *ε*-matches from position 4 to position 31 is not an *ε*-match: the error rate is 3/28 > 0.1. Furthermore, the intersection of the two *ε*-matches is with 19 columns too short to be an *ε*-match.

A pairwise local alignment of length *n* is a sequence of *n* match, insertion, deletion, and substitution columns. In our approach deletion, insertion, and substitution columns are all treated equally. Hence, we will call these columns *error* columns. The number of error columns is the edit or Levenshtein distance of a local alignment. Normalizing this distance by dividing it by the length of the local alignment, we obtain an *error rate.* An *ε-match* is a local alignment that has an error rate of at most *ε* > 0 and length *n* ≥ *n*_0_. Fig. 1 shows two examples of *ε*-matches as segments of a longer local alignment.

**Figure 2:**
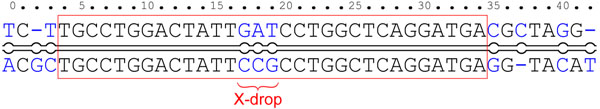
**X-drop**. An alignment containing an X-drop for *X* = 3. In this example, an *ε*-match with *ε* = 0.1 and *n*_0_ = 20 (indicated by the box) spans the X-drop.

**Figure 3:**
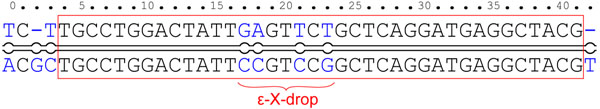
***ε*-X-drop.** An alignment containing an *ε*-X-drop for *X* = 3 and *ε* = 0.1. To reach an *ε*-X-drop with a score drop-off of at least , a fourth error column is necessary in this example because of the positively scoring matches in between the errors.

The notion of an *X – drop* to delineate local alignments from each other is well established by Miller and coworkers in the context of similarity alignments [18,26,27]. An *X-drop* within an alignment, where *X* > 0 is given, is a region of consecutive columns with a total score of *–X* or less. In other words, it is a region where the score drops by *X* or more. In Fig. 2 we display an example in which we score error columns by –1. The X-drop is a very intuitive way to model local dissimilarity and hence we choose to adopt the concept for our model of local similarity. Since we address the computation of *ε*-matches in this paper we propose the following scoring scheme that depends on the error rate *ε*: We score a match by +1 and an error by *p* = *–*1/*ε* + 1. In addition, we adjust the score drop-off parameter by multiplying it with the negative of the error penalty –*p* such that the user specified parameter *X* still corresponds to the number of errors in a match-free X-drop region and is easy to grasp for the user (Fig. 3). To emphasize the difference from the usual understanding of an X-drop we call this weighted X-drop an *ε-X-drop.*

The goal of this work is to find *ε*-matches of two sequences without *ε*-X-drops. Often, *ε*-matches overlap to a large extent (see Fig. 1 for an example), or segments of *ε*-matches are themselves *ε*-matches (e.g. we obtain an *ε*-match by removing a column from one end of the *ε*-matches in Fig. 1). Thus, the number of *ε*-matches can be very large with very redundant similarity information. We handle this issue by only identifying the longest *ε*-match of one location: If two *ε*-matches overlap, we output only the longer one unless the overhanging part of the shorter *ε*-match still has a minimal length of *n*_0_. In that case we output both complete *ε*-matches. Thus, for the example in Fig. 1 we only output one *ε*-match. We say that such an *ε*-match is *maximal.*

We now present an algorithm to compute exactly *all* maximal *ε*-matches without *ε*-X-drop. Our algorithm runs in two phases: filtering and verification. The filtering phase implements the SWIFT algorithm [28], a very efficient full-sensitivity filter for *ε*-matches. Note that SWIFT is a *filter* algorithm and does not output a list of *ε*-matches. While it guarantees not to miss any maximal *ε*-match and hugely reduces the search space, a verification phase is necessary to identify false positive hits of the filter. Furthermore, verification is needed to determine the exact start and end positions of maximal *ε*-matches. We have developed a verification strategy that runs in five steps: *ε*-core identification, *ε*-X-drop core filter, *ε*-X-drop extension, identification of maximal *ε*-matches, and filtering of overlapping matches. Verification may stop after any of these steps if it is clear that we will not identify a new *ε*-match without *ε*-X-drop. The strategy guarantees to find all maximal *ε*-matches without *ε*-X-drop.

A similar two-step algorithm that consists of SWIFT filtering and subsequent verification is implemented in the read mapper RazerS [14]. The difference to RazerS is, however, that we are looking for *ε*-matches that are local in both sequences whereas read mappers compute semi-global alignments, i.e align the full read sequence to a reference. RazerS uses a slightly modified SWIFT filtering, and the verification is much simpler since the length of the final alignments is preset by the read length.

### Filtering phase

The SWIFT algorithm proposed by Rasmussen et al. is an efficient *q*-gram based filter to detect potential *ε*-match regions between two sequences. It is based on the *q*-gram lemma [29,30]. This lemma states that every alignment of length *n* with *k* error columns contains at least *T*(*n*, *k*, *q*) := *n* + 1 – *q*(*k* + 1) *q*-hits, substrings of *q* consecutive match columns. Considering the dotplot of two sequences, every *q*-hit corresponds to a diagonal stretch of matches with length *q.* Obviously, all *q*-hits of an alignment with *k* errors can cover at most *k* + 1 different diagonals in the dotplot.

Rasmussen et al. proved that for any given *ε* and *n*_0_ there exist *w*, *q*, *e* and *τ* such that every *ε*-match contains *τ q*-hits that reside in a *w* × *e* parallelogram. A *w* × *e* parallelogram is the intersection of *e* + 1 consecutive diagonals and *w* + 1 consecutive columns in the dotplot.

To detect *w* × *e* parallelograms with *τ q*-hits in the dotplot, the SWIFT algorithm slides from left to right over one sequence and searches overlapping *q*-grams in a *q*-gram index of the other sequence. Found *q*-hits are counted in bins of Δ + *e* consecutive diagonals whose first diagonal is a multiple of Δ. As adjacent bins share e diagonals, every *w* × *e* parallelogram is contained in one bin. Every bin contains a *q*-hit counter and represents the parallelogram with columns bounded by the leftmost and rightmost contained *q*-hit. If a *q*-hit is found that is at most *w – q* columns apart from the rightmost *q*-hit, the parallelogram is extended. Otherwise it is closed and a new one starting at the found *q*-hit is opened as the two hits cannot be part of the same *w* × *e* parallelogram. A closed parallelogram whose bin counter has reached *τ* is output as a *SWIFT hit* and verified as described in the following section.

**Figure 4:**
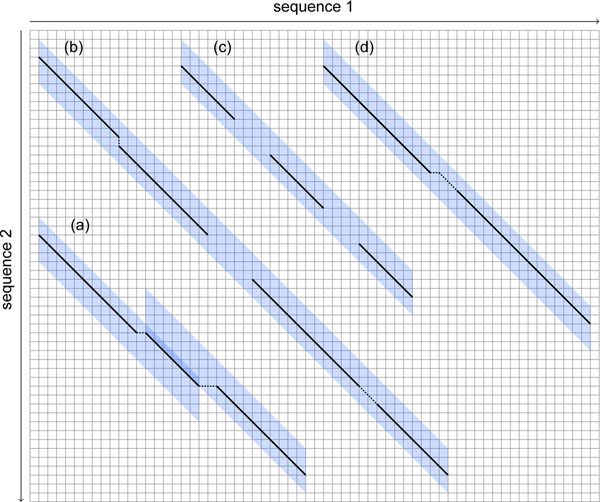
**Example SWIFT hits**. Example SWIFT hits for *n*_0_ = 20, *ε* = 0.1, and *q* = 6 (= *s^min^*). Accordingly, *w* = 20, *τ* = 3, and e = 2. SWIFT searches parallelograms that contain at least *τ* = 3 *q*-gram hits by streaming over sequence 1 and searching common *q*-grams in sequence 2. Subfigure (a) shows an *ε*-match that results in two SWIFT hits and the *ε*-match is longer than both of the two hits. (b) shows a SWIFT hit that contains two *ε*-matches and (c) shows a false positive SWIFT hit induced by three separated *q*-gram hits. (d) shows a SWIFT hit that contains an *ε*-match with a 3-drop.

Fig. 4 shows examples for SWIFT hits containing either a subalignment of an *ε*-match, whole *ε*-matches, no *ε*-match or an *ε*-match with an X-drop.

### Verification phase

Fig. 4 demonstrates that the output of the filtering phase is not yet a list of *ε*-matches, although the SWIFT algorithm hugely reduces the search space. SWIFT hits may contain one or more *ε*-matches, but may as well be false positive and contain no *ε*-match. Some SWIFT hits may overlap and contain the same or parts of the same *ε*-match. Further, they may be much longer than a contained *ε*-match, or they may cover only parts of an *ε*-match. Therefore, we have developed the following verification strategy.

We start verifying SWIFT hits by identifying a segment of an *ε*-match that overlaps with the SWIFT hit. We call such a segment an *ε-core.* We guarantee not to miss any *ε*-match by identifying all *ε*-cores contained in a SWIFT hit. *ε*-cores will then serve as starting points for extension, possibly beyond the ends of SWIFT hits. Finally, we cut the longest *ε*-matches from extended *ε*-cores and remove overlapping *ε*-matches.

#### Definition and existence of *ε*-cores

Under the simple scoring scheme where a match scores +1 and an error *p* = *–*1/*ε* + 1, we define an *ε*-core of an *ε*-match as a segment with a score of at least:

where *n*_0_ is the minimal length of an *ε*-match and  is the next larger length that allows one more error than *n*_0_.

In the following two lemmata we prove the correctness of our approach that starts verification from *ε*-cores.

**Lemma 1. ***Every ε-mαtch contains at least one ε-core.*

**Figure 5:**
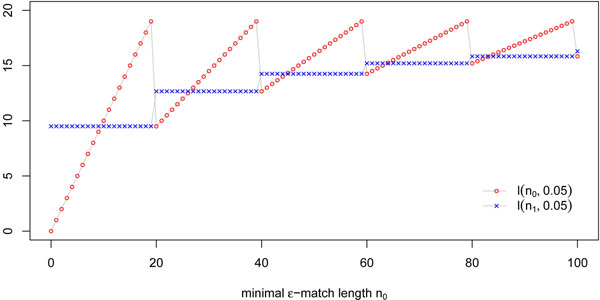
**Sawtoothed function.** Plot of the sawtoothed function  for *n*_0_ and  with *ε* = 0.05.

*Proof.* is the maximal number of errors in an *ε*-match of length *n*. Thus, the number of matching positions in an *ε*-match of length *n* is at least . These  matching positions can be split by the errors of the *ε*-match into at most  error-free segments. If the errors are spread evenly over the *ε*-match, at least one of the error-free segments has length ≥ *l*(*n*, *ε*), where . Some thought reveals that any other distribution of the errors would result in at least one longer error-free segment. Therefore, an *ε*-match of length *n* contains at least one *ε*-core of length ≥ *l*(*n*, *ε*)*.* Unfortunately, *l*(*n*, *ε*) is a sawtooth function, i.e. *l*(*n*, *ε*) is not monotonically increasing in *n* (Fig. 5). Hence, one cannot use *l*(*n*_0_, *ε*) as a bound for all *n* ≥ *n*_0_. The function drops to a minimum at each point . However, it is easy to confirm that the minima are strictly increasing, i.e. each successive minimum of the sawtooth is higher than the previous one. Therefore, the smallest value of *l*(*n*, *ε*) over all *n* ≥ *n*_0_ is the minimum of *l*(*n*_0_, *ε*) and *l*(*n*_1_, *ε*). This is exactly the definition of *s*^min^, i.e. an *ε*-match contains at least one error-free segment of length *s^min^* which is an *ε*-core. □

Lemma 1 settles the existence of an *ε*-core for every *ε*-match. Unfortunately, the SWIFT filter only guarantees to report SWIFT hits that *overlap* a part of each *ε*-match. Hence, in principle, the SWIFT hit could not contain an *ε*-core, which in turn could make our algorithm miss the *ε*-match. In the next lemma we will show that for a certain value of the parameter *q* this is never the case.

**Lemma 2. ***The intersection of a SWIFT hit with an ε-match contains at least one ε-core if q* := *s^min^.*

*Proof.* By definition, the intersection of a SWIFT hit with an *ε*-match contains at least *τ q*-grams interspersed by at most *e* errors. Therefore, every SWIFT hit contains at least one segment of at least  consecutive *q*-grams. The length of this segment is at least . Because *τ* > 0 and e ≥ 0 the first summand  is greater or equal one, so we obtain . Thus, if we set *q* := *s^min^* every SWIFT hit contains at least one segment of length *s^min^*, which is an *ε*-core. □

#### Step 1: *ε*-core identification

In our verification strategy, we identify *ε*-cores by applying a banded version of the Waterman-Eggert local alignment algorithm [31]. The original algorithm computes all non-overlapping local alignments that reach a specified minimal score under a certain scoring scheme by dynamic programming (DP). We use the scoring scheme that scores matches by +1 and errors by *p* = *–*1/*ε* + 1 and set the minimal score to *s^min^.* In our version, we reduce running time and space requirements of the algorithm by banding the computation of the DP matrix according to the parallelogram shape of SWIFT hits (see Fig. 4, only the shaded parts of the alignment matrix need to be computed). Thus, per *ε*-core there is a maximum running time of  where *w′* is the length and *e* the width of the corresponding SWIFT hit.

**Figure 6:**
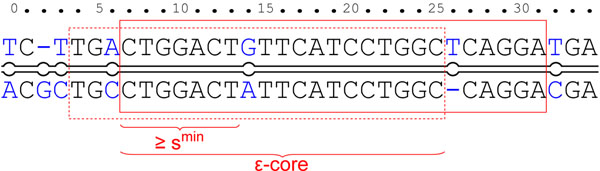
**Extended *ε*-core**. An *ε*-match extended by one error column and  match columns, is still an *ε*-match. Therefore, an *ε*-core should include such an extension, since all maximal *ε*-matches at this location will include it. In this example where *ε* = 0.1 and *n*_0_ = 20 we see an error-free segment of length 7 ≥ 6 = *s^min^* that can be extended to an *ε*-core of length 19 including one error.

Since the Waterman-Eggert algorithm reports only non-overlapping local alignments one may think that some *ε*-cores will not be identified because they are hidden by longer local alignments that reach beyond the end of an *ε*-match. However, this can only be for non-maximal *ε*-matches since we have chosen the scoring parameters such that *ε*-cores extended by additional local alignment columns only have higher scores if the additional columns have themselves an error rate of at most *ε* (Fig. 6). This is why all maximal *ε*-matches will also include the additional columns, i.e. no local alignment will reach beyond the end of an *ε*-match.

#### Step 2: *ε*-X-drop core filter

The second step of our verification strategy is a filter for *ε*-X-drops in the *ε*-cores. In the previous step we ignored *ε*-X-drops in *ε*-cores. If now one of our *ε*-cores contains an *ε*-X-drop, the *ε*-core should be divided into two cores in order to remove the *ε*-X-drop. Similarly, if an *ε*-core contains more than one *ε*-X-drop, the *ε*-core should be divided into even more cores.

For this decomposition of the *ε*-cores, we apply the post-processing algorithm from Zhang et al. [27] with the same scores and penalties for matching and error positions as in our *ε*-core identification step. The worst-case running time of this algorithm is linear in the length of the *ε*-core.

Possibly, we obtain more than one *ε*-core after this step, but in any case the following extension step has to be conducted only to the left and right of the original non-decomposed *ε*-core. If we started with an *ε*-core with more than one *ε*-X-drop, we can skip the following extension step for the middle parts, since the extension algorithm would run immediately into the previously detected *ε*-X-drops.

#### Step 3: *ε*-X-drop extension

The goal of the extension step is to obtain a region that spans all *ε*-matches without *ε*-X-drop containing the *ε*-core. In this region, the *extended ε-core*, we can then look for the maximal *ε*-match. Clearly, we can discard extended *ε*-cores that are shorter than *n*_0_.

For extension we apply the gapped extension algorithm by Zhang et al. [19] with the *ε*-adjusted scoring parameters as before. This algorithm is a score-only algorithm, i.e. it reports only the score and the sequence positions of the maximal extension but not the precise alignment. However, it reports the maximal and minimal diagonal of the alignment matrix (a band) that needs to be computed when looking for the precise alignment. We will determine the alignment in the next step of the verification strategy together with the exact begin and end positions of the maximal *ε*-match.

It is hard to do an informative running time analysis for this step. In theory, the dynamic programming algorithm could fill big parts of the alignment matrix. However, for very similar sequences only a narrow diagonal stretch will be filled, and for very distinct sequences we will soon reach an X-drop and stop. Still, we can estimate the running time by  where *L* is the length of the extension and *b* is the width of the band.

It is easy to confirm that if an *ε*-core is part of an *ε*-match without *ε*-X-drop, then the extended *ε*-core spans this *ε*-match without *ε*-X-drop.

#### Step 4: identification of maximal *ε*-matches

**Figure 7:**
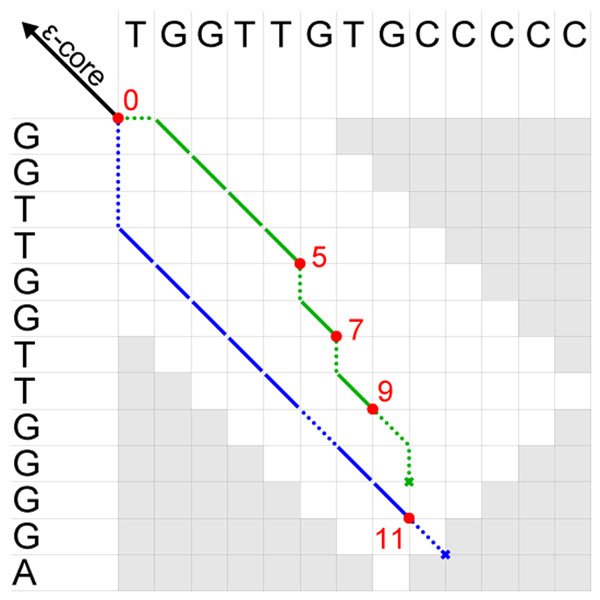
**Extension traces of an *ε*-core.** Alignment matrix with two possible traces of the extension of an *ε*-core. Depending on the length of the extension (red numbers), we obtain the lowest error rates by aligning different sequence positions to each other. For an extension length of 5, 7, and 9 it is better to follow the upper trace, whereas for a length of 11 the lower trace has fewer errors. For all other lengths there is a longer trace with the same number of errors, and therefore it is not necessary to consider those lengths while looking for maximal *ε*-matches.

The remaining task is to determine the longest alignment in the extended *ε*-core that has an error rate of at most *ε.* More precisely, we are looking for the longest extension to the left and to the right of the *ε*-core such that the complete alignment has an error rate of at most *ε.* The maximal error rate (i.e. the number of errors and length) that we can allow for the extension to the left depends not only on the error rate of the *ε*-core but also on the error rate of the extension to the right, and vice versa. Therefore, we cannot determine the lengths of the right and left extension separately. Furthermore, depending on the length of the extension the optimal trace through the alignment matrix can differ (Fig. 7). Our suggestion is to compute for all possible extension lengths the optimal end position of a trace in the alignment matrix, then to determine the optimal lengths of the right and left extension, and lastly to carry out the traceback. The details of these three steps are described in the following.

The computation of the optimal end position of a trace for all possible extension lengths can be done along with the computation of the alignment matrix. Unfortunately, two traces of different lengths may end in the same column of the alignment matrix. For this reason, it is necessary to compute the alignment matrix using an algorithm for normalized alignment score [32,33], which in addition iterates over all alignment lengths. As already mentioned, the alignment matrix can be banded by the minimal and maximal diagonal from the seed extension algorithm.

Along with the alignment matrix computation we check and if necessary update in each iteration step a list *bestEnds* with the best alignment matrix entry for the corresponding extension length. Each entry consists of the length and score (or number of errors) of the alignment, and coordinates in the alignment matrix. This list can afterward be reduced to a smaller set of possible lengths using the following observation: The position before the start and the position behind the end of the sought *ε*-match is an error, otherwise the *ε*-match would not be maximal. Hence, we only need to keep those list entries for lengths *l* where the score difference of *bestEnd*[*l*] and *bestEnd*[*l* + 1] is smaller than the score of a match. As a result we obtain two lists with possible traceback starting points, one for start positions of the *ε*-match obtained from the left extension, and one for end positions obtained from the right extension.

On these lists we then apply the following exhaustive search algorithm that iterates over combinations of possible start and end positions: We start with the leftmost possible start position and iterate over possible end positions from right to left. We continue with the next possible start position and restart with the rightmost possible end position as soon as the segment between the current start and end position has an error rate of at most *ε* (update currently longest *ε*-match), or if this segment is shorter than the minimal *ε*-match length or our currently longest *ε*-match (do not update currently longest *ε*-match). The algorithm stops when the segment between the current start position and the rightmost possible end position is shorter than the minimal *ε*-match length or the currently longest *ε*-match. Using this strategy we cannot miss the longest *ε*-match without *ε*-X-drop if the *ε*-core is part of one.

In case there is another maximal *ε*-match containing the *ε*-core, we have to recurse this search twice with the lists reduced by the following entries: All start positions before the start position of the longest *ε*-match and all end positions that are smaller than *n*_0_ added to the end position of the longest *ε*-match; and all start positions before the start position of the longest *ε*-match minus *n*_0_ and all end positions behind the end position of the longest *ε*-match.

As a last step, we have to look up the coordinates for the optimal extensions in the *bestEnd* lists and start traceback from these positions in the alignment matrices. The *ε*-core extended by the resulting alignments is a maximal *ε*-match that contains the *ε*-core.

The running time for computing the alignment matrix using a normalized alignment score is in  where *b* is again the width of the band and *L* is the length of the extension. Dropping the normalization of the alignment score reduces running time by a factor of *L.* Determining the optimal start end end position in the left extension of length *L*_1_ and right extension of length *L*_2_ takes  time per *ε*-match, and finally, the traceback takes time linear in the length of the final *ε*-match.

#### Step 5: removal of largely overlapping *ε*-matches

An *ε*-match identified during the verification phase is the longest that contains one specific *ε*-core but it is not necessarily maximal in that it does not overlap with a longer *ε*-match. In addition, an *ε*-match containing two *ε*-cores will be identified twice. To ensure that we output each *ε*-match only once and also only maximal *ε*-matches, this last step is necessary.

We remove overlapping *ε*-matches by sorting the *ε*-matches by their begin position in one sequence and pairwisely comparing here overlapping matches further. If two *ε*-matches are found to be identical, one is discarded. Also, if the shorter of the two *ε*-matches has no unique part of length *n*_0_, this *ε*-match is discarded. The running time of this last step is dominated by sorting the *ε*-matches, i.e. it is in , where *M* is the number of *ε*-matches before removal.

**Theorem.***Let M be the set of maximal ε-matches without ε-X-drop between two sequences. Then the algorithm that uses SWIFT for filtering and the described strategy for verification will detect exactly all ε-matches in M.*

*Proof.* The SWIFT filter algorithm guarantees to report at least one overlapping SWIFT hit for every *ε*-match of the input sequences. The first step of the verification strategy detects all *ε*-cores in SWIFT hits. Apply Lemma 1 to prove that every *ε*-match contains an *ε*-core. According to Lemma 2, one of the *ε*-cores of every *ε*-match is contained in a SWIFT hit. Thus, for every *ε*-match in *M* the first verification step identifies at least one *ε*-core.

Let *C′* be the set of *ε*-cores identified during the first step, and let *C* ⊆ *C′* be the subset of *ε*-cores that are part of an *ε*-match in *M.* Since none of the *ε*-matches in *M* contain an *ε*-X-drop, the local alignment obtained after *ε*-X-drop extension (Step 3) of an *ε*-core *c* ∈ *C* spans the corresponding *ε*-match.

By cutting the extended *ε*-cores as described in Step 4, we eventually end up with a set of *ε*-matches *M****′*** that each contains a certain *ε*-core. Step 4 also guarantees that per *ε*-core no longer *ε*-match exists than the *ε*-match in *M′*. Therefore, after removal of overlapping *ε*-matches (Step 5), our set of *ε*-matches contains exactly all maximal *ε*-matches without *ε*-X-drop. □

## Results and discussion

We have implemented the algorithmic pipeline in the program STELLAR following exactly the above described steps with one exception: To improve running time, STELLAR computes the alignment matrix during the identification of the longest *ε*-match with unnormalized alignment score. The following results show that this has in practice no effect on the sensitivity.

**Figure 8:**
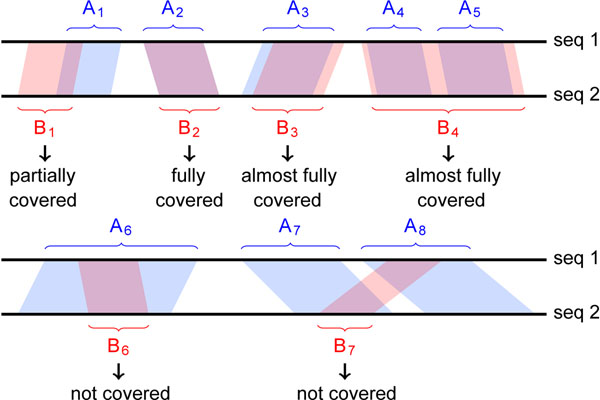
**Match coverage.** A set of matches *A* = {*A*_1_, …, *A*_8_} that are to be compared to a set of reference matches {*B*_1_, …, *B*_7_}*.* We say that a match *B_i_* is covered by the matches from *A* if at least 10% of the alignment columns agree between *B_i_* and any match from *A.*

We tested STELLAR on simulated and on real genomic data and compared its performance to BLAST [18], LASTZ as the replacement of BLASTZ [21], BLAT [20], BWT-SW [22], and Smith-Waterman alignments obtained with SSEARCH from the FASTA package [16]. In addition, we ran BLAST with a more sensitive parameter setting: According to Lemma 1, every *ε*-match contains a seed of length *s^min^*, and therefore, we set BLAST’s word-size parameter to the corresponding *s^min^* computed in STELLAR. To demonstrate the differences between the programs we compared speed and sensitivity on all data sets. Running times were measured on a 2.66 GHz Intel Xeon X5550 with 72 GB of RAM running Linux. Running times of BWT-SW include pre-processing of the database sequence. In all test runs STELLAR needed less than 1 GB of RAM, so we omit further details of memory usage. As a measurement for sensitivity we computed the percentage of matches from a reference set that were sufficiently covered by matches from the respective program. We say that matches that are covered by less than 10% are missed (Fig. 8). This is a very loose criterion which is in favor of the compared programs.

### Simulated sequences

**Figure 9:**
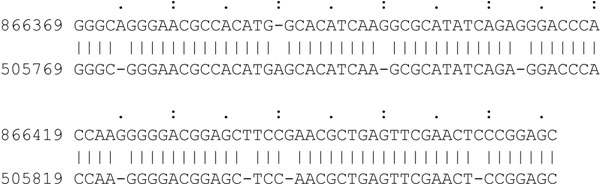
***ε*-match in simulated data.** An example *ε*-match from the simulated sequences of length 1 Mb and *ε* = 10%. This *ε*-match with an E-value of 7 × 10^–26^ is only found by STELLAR, BWT-SW, and SSEARCH, but by none of the other programs.

To demonstrate STELLAR’s gain of sensitivity in comparison to other programs, we used simulated data sets. The advantage here is that the reference set of local alignments for the computation of the sensitivity is given. We simulated random sequences with uniformly distributed characters from the alphabet {A, C, G, T}. In addition, we simulated random local alignments of length 50-200 bp and inserted errors at different rates into the alignments. In order to see at what error rates the programs start missing local alignments, we created a first data set where 500 such local alignments with an error rate of 0%, 2.5%, 5%, 7.5%, or 10% were inserted into a sequence of length 1 Mb at random positions. A second simulation was conducted to assess the effect of the sequence length. Here, sequences of lengths 1 kb, 10 kb, 100 kb, 1 Mb, and 10 Mb were simulated containing local alignments with error rates between 0 and 10%. On these data sets we ran the above mentioned programs, with STELLAR’s error rate parameter set accordingly and the minimal match length set to 50. The results are shown in Tables 1 and 2. Table 1 demonstrates that STELLAR, in particular for the higher error rates, outperforms the other programs. SSEARCH has full sensitivity as expected but is very slow. We confirmed that with an E-value cutoff of 0.01 it does not detect any other than the inserted local alignments. BWT-SW reports all local alignments, too, but is still much slower than STELLAR. For BLAST and LASTZ the number of missed matches is low for very low error rates but increases with higher error rates. This implies that one can benefit the most from STELLAR when comparing closely related sequences that still have significant differences. As an example, Fig. 9 displays one *ε*-match that only STELLAR, BWT-SW, and SSEARCH identify. BLAST is the fastest of all programs, though only with default parameters and lower sensitivity. BLAT constantly misses around 30% of all matches. We assume that the reason for BLAT’s bad performance is that it was originally designed for the comparison of many short sequences (ESTs or reads) against one long sequence and not for the comparison of two long sequences. Table 2 supports this assumption, as the number of matches missed by BLAT is low for the 1 kb – 100 kb sequences but increases up to almost 70% for sequences of length 10 Mb. In contrast, the sensitivity of BLAST and LASTZ seems not to be affected by different sequence lengths. STELLAR is in general faster than BWT-SW, BLAT, and LASTZ with one exception – the 10 Mb sequences. This indicates already a limitation of STELLAR on very long sequences with high error rates. The SWIFT filter has a lower specificity for high error rates and generates very many SWIFT hits. As a result, many verification steps are necessary, which leads to an increase in running time. However, STELLAR is faster than the sensitive BLAST for the 10 Mb sequences and also for high error rates. The sudden increase in running time for the sensitive BLAST at higher error rates (Table 1) is due to a much lower *s^min^* for *ε* = 10%.

**Table 1:** Running times and sensitivity on simulated sequences containing local alignments of different error rates

error rate	0%		2.5%		5%		7.5%		10%	
	time	missed	time	missed	time	missed	time	missed	time	missed
SSEARCH	133:17 h	0.00%	132:55 h	0.00%	133:24 h	0.00%	132:25 h	0.00%	132:52 h	0.00%
STELLAR	2.96 s	0.00%	3.30 s	0.00%	3.61 s	0.00%	3.91 s	0.00%	4.44 s	0.00%
BWT-SW	16.59 s	0.00%	16.63 s	0.00%	16.53 s	0.00%	16.29 s	0.00%	16.24 s	0.00%
BLAST	0.25 s	0.00%	0.26 s	0.16%	0.26 s	5.36%	0.25 s	17.00%	0.24 s	38.80%
BLAST*	0.25 s	0.16%	0.26 s	0.00%	0.28 s	0.04%	0.51 s	0.28%	14.99 s	2.60%
LASTZ	6.49 s	0.00%	6.48 s	0.72%	6.22 s	5.56%	5.86 s	12.68%	5.23 s	24.92%
BLAT	14.30 s	29.36%	11.52 s	29.64%	14.06 s	28.88%	14.71 s	31.44%	14.66 s	34.32%

Sequences have a length of 1 Mb and contain local alignments of lengths between 50 and 200 bp. The simulations were repeated five times, the displayed values are the average of all runs except for SSEARCH which was run only once. Sensitivity is measured by the percentage of missed local alignments (Fig. 8). BWT-SW, BLAST, BLAT, and LASTZ were run with default parameter settings. BLAST* stands for a more sensitive run of BLAST with the word size parameter set to *s^min^*, i.e. the minimal length of an *ε*-core (see text for details).

**Table 2:** Running times and sensitivity on simulated sequences of different lengths

seq length	1 kb		10 kb		100 kb		1 Mb		10 Mb	
	time	missed	time	missed	time	missed	time	missed	time	missed
SSEARCH	2.45 s	0.00%	259.5 s	0.00%	7:16 h	0.00%	136:16 h	0.00%	–	–
STELLAR	1 ms	0.00%	5 ms	0.00%	0.07 s	0.00%	4.36 s	0.00%	782.46 s	0.00%
BWT-SW	–	–	787 ms	0.00%	1.40 s	0.00%	16.33 s	0.00%	508.45 s	0.00%
BLAST	4 ms	14.00%	8 ms	6.00%	0.03 s	11.40%	0.25 s	13.40%	3.34 s	12.64%
BLAST*	6 ms	0.00%	13 ms	0.00%	0.16 s	0.00%	14.75 s	0.60%	2266.64 s	1.46%
LASTZ	9 ms	10.00%	59 ms	2.00%	0.56 s	7.80%	5.99 s	9.40%	116.26 s	9.12%
BLAT	25 ms	0.00%	39 ms	0.00%	0.40 s	1.00%	15.24 s	33.00%	384.79 s	69.30%

Sequences contain *a* = [length/2000] local alignments with a maximal error rate of 10% and lengths between 50 and 200 bp. The simulation of the 1 kb sequences was repeated 50 times, the simulation of the 10 kb and 100 kb sequences ten times. The displayed values are the average of all runs except for SSEARCH which was run only once. Sensitivity is measured by the percentage of missed local alignments (Fig. 8). BWT-SW, BLAST, BLAT, and LASTZ were run with default parameter settings. BLAST* stands for a more sensitive run of BLAST with the word size parameter set to *s^min^*, i.e. the minimal length of an *ε*-core (see text for details).

### Genomic sequences

We downloaded the assembled genomes of *Drosophíla melanogaster* (release 5.26) and *Drosophíla pseudoobscura* (release 2.14) from FlyBase [34]. We selected chromosome arm 2L from *D. melanogaster* (~23.5 Mb) and group 3 from the chromosome 4 assembly of *D. pseudoobscura* (~11.7 Mb) for our test runs. Unfortunately, this data set is too big to compute local alignments with SSEARCH, and since BLAST performs better than BLAT and LASTZ on the simulated data we chose to compare STELLAR on the genomic data only to BLAST. We expect BLAST to compute very short alignments with low error rates and some long alignments with higher error rates that do not fulfill the minimal length or error rate criterion for *ε*-matches, and hence STELLAR will not find them. Therefore, to double-check STELLAR’s sensitivity, we filter all *ε*-matches from the set of BLAST hits. Additonally, some of the longer BLAST hits may contain valid *ε*-matches that we extract and add to the set of filtered BLAST hits.

Results for STELLAR are shown in Table 3 and results for BLAST in Table 4. STELLAR identifies for example 345 *ε*-matches with an error rate of 10% and a minimal match length of 200. As expected, these cover all of the *ε*-matches filtered from the set of BLAST hits. In contrast to the simulated sequences, the running time increases significantly with a higher error rate or lower minimal length. This can be explained with the filter algorithm being more specific for higher minimal lengths and low error rates as already mentioned above. With less specific filtering, many more SWIFT hits need to be verified resulting in a higher running time. In the future we might be able to reduce this effect by parallelization.

**Table 3:** Results of STELLAR on drosophila chromosomes

error rate	min. length	running time	num. of *ε*-matches	overlap BLAST*^a^*
10%	100	7777 s	3911	100%
10%	200	1566 s	345	100%
5%	200	21 s	44	100%

We used chromosome arm 2L from *D. melanogaster* (~23.5 Mb) and group 3 of chromosome 4 from *D.pseudoobscura* (~11.7Mb). STELLAR was run with the X-drop parameter set to 20.

*^a^* Percentage of covered local alignments from accordingly filtered BLAST output.

**Table 4:** Results of BLAST on drosophila chromosomes

word size	running time	num. of hits	overlap STELLAR 200*^b^*	overlap STELLAR 100*^b^*
default	9 s	9504	95.1%	89.6%
8	2175 s	29597	100.0%	99.7%

We used chromosome arm 2L from *D. melanogaster* (~23.5 Mb) and group 3 of chromosome 4 from *D.pseudoobscura* (~11.7Mb).

*^a^* Percentage of covered STELLAR matches (error rate 10%, minimal length 200 or 100).

**Figure 10:**
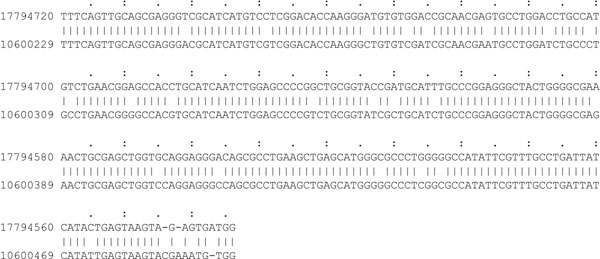
**ε-match between drosophila sequences**. An example *ε*-match from the drosophila sequences with *ε* = 10% and *n*_0_ = 200. This *ε*-match with an E-value of 6 × 10^–84^ is not found by BLAST with default parameters.

BLAST with default settings is again the faster program, but misses 17 *ε*-matches of minimal length 200. One of these matches is displayed in Fig. 10. When we change the word size parameter such that BLAST is able to identify all *ε*-matches of minimal length 200, it is slower than STELLAR. STELLAR with minimal length 100 and error rate 10% is slowest in all tested settings, but identifies 408 *ε*-matches that BLAST with default parameters does not find, and 13 *ε*-matches that even the more sensitive setting of BLAST does not find though these *ε*-matches have an E-value of 6.1 x 10^–23^ or lower.

## Conclusions

We presented STELLAR, an algorithm to compute *all* local alignments of a minimal length according to a clear quality definition using the established measures *error rate* and *X-drop.* STELLAR brings exact local alignments to the community at the speed of heuristic state-of-the-art tools like BLAST, BLAT, or LASTZ. In addition, our experiments show that our effort is worthwhile since the heuristic tools miss up to about a third of the matches using simulated and real genomic data. Compared to its closest competitor, BWT-SW, it is in most benchmarks faster and offers with the *X-drop* parameter a possibility to exclude local alignments with bad regions. A limitation of STELLAR is that only *ε*-matches up to a certain error rate can be computed since the filtering phase loses specificity with increasing error rate. Therefore, for longer and less similar though significant local alignments BLAST remains more appropriate.

As an outlook another relatively new application for local alignments that has emerged with the advent of cheap next-generation sequencing should be mentioned. Standard read mapping programs [8-14] usually can only map entire reads to the reference. With the increasing read length, there will be more reads that span breakage points, e.g. translocations, gene fusions, or splice junctions. The application of an efficient and exact local alignment program could be one way to successfully map such reads and detect variation [15]. Application of STELLAR is especially promising in that it uses the error rate for sensitivity control, an established criterion for read mappers. With a downstream chaining procedure of the partial read matches detected by STELLAR, it may then be possible to detect even multiple splits of reads. Hence, for finding local alignments in the tested range of error rates STELLAR could replace the heuristic tools.

## Competing interests

The authors declare that they have no competing interests.
